# Enhancing the Potential of Polymer Composites Using Biochar as a Filler: A Review

**DOI:** 10.3390/polym15193981

**Published:** 2023-10-03

**Authors:** Mohamed Aboughaly, Amin Babaei-Ghazvini, Piyali Dhar, Ravi Patel, Bishnu Acharya

**Affiliations:** Department of Chemical and Biological Engineering, University of Saskatchewan, 57 Campus Drive, Saskatoon, SK S7N 5A9, Canada; ust024@mail.usask.ca (M.A.); amin.babaei@usask.ca (A.B.-G.); yzg859@mail.usask.ca (P.D.); patel.ravi@usask.ca (R.P.)

**Keywords:** biochar, carbonaceous fillers, polymer properties, carbonization, pyrolysis, carbonization

## Abstract

This article discusses the scope biochar’s uses; biochar is a sustainable organic material, rich in carbon, that can be synthesized from various types of biomass feedstock using thermochemical reactions such as pyrolysis or carbonization. Biochar is an eco-friendly filler material that can enhance polymer composites’ mechanical, thermal, and electrical performances. In comparison to three inorganic fillers, namely carbon black, carbon nanotubes (CNT), and carbon filaments, this paper explores the optimal operating conditions for regulating biochar’s physical characteristics, including pore size, macro- and microporosity, and mechanical, thermal, and electrical properties. Additionally, this article presents a comparative analysis of biochar yield from various thermochemical processes. Moreover, the review examines how the surface functionality, surface area, and particle size of biochar can influence its mechanical and electrical performance as a filler material in polymer composites at different biochar loads. The study showcases the outstanding properties of biochar and recommends optimal loads that can improve the mechanical, thermal, and electrical properties of polymer composites.

## 1. Introduction

The global polymer industry’s annual value is estimated to reach USD 600 billion [1]. The projected growth rate of the industry is expected to contribute towards the accumulation of 12,000 Mt of plastic wastes in landfills by 2050 [2]. This would negatively impact the natural environment. The development of polymer composites with bio-based additives is essential to mitigate the impact of the use of non-renewable petroleum-based materials on the environment. There is a growing demand for polymer composites with improved properties, such as density, stiffness, strength, and lower cost, due to their various applications in many industries, including the aerospace, automotive, packaging, and pharmaceutical industries. Thermoplastic polymer composites form 23% of global matrix composites due to their ability for reheating, re-melting, and reshaping. Thermoset polymer composites are the most common due to their great flexibility in product designs, improved structural integrity, and molding ability with different tolerances. However, these composites may suffer from drawbacks such as low ductility, high voids, limited thermal stability, and low flame retardancy. The addition of various fillers to polymer composites can help to overcome these limitations [1]. Biochar is a promising, environmentally friendly, low-cost, and sustainable filler material [2,3,4,5] which is produced from biomass pyrolysis or carbonization. It outperforms traditional fillers such as carbon black, carbon nanotubes, and carbon filaments. Biochar has certain properties, such as high hydrophobicity, which improve its compatibility with the polymer matrix and render a higher thermal stability compared to alternative filler materials [2,3,4]. This paper highlights the impact of biochar at different loads on the mechanical, electrical, and thermal performances of polymer composites. A comparison is made between different carbon-based materials as fillers in polymer composites [3,4,5].

## 2. Production, Properties, and Potential Applications of Biochar in Composites

Lignocellulosic biomass, an abundant and renewable resource, is mainly composed of cellulose, hemicellulose, and lignin. It is a promising alternative to fossil resources and a potential feedstock for producing biofuels, bio-based chemicals, and materials including biochar [6]. Lignocellulosic materials are found in all vascular plants, commonly between and within the cells, as well as within the cell walls. Biochar is synthesized from biomass via thermochemical processes such as pyrolysis, carbonization, and gasification [7,8].

**Figure 1:**
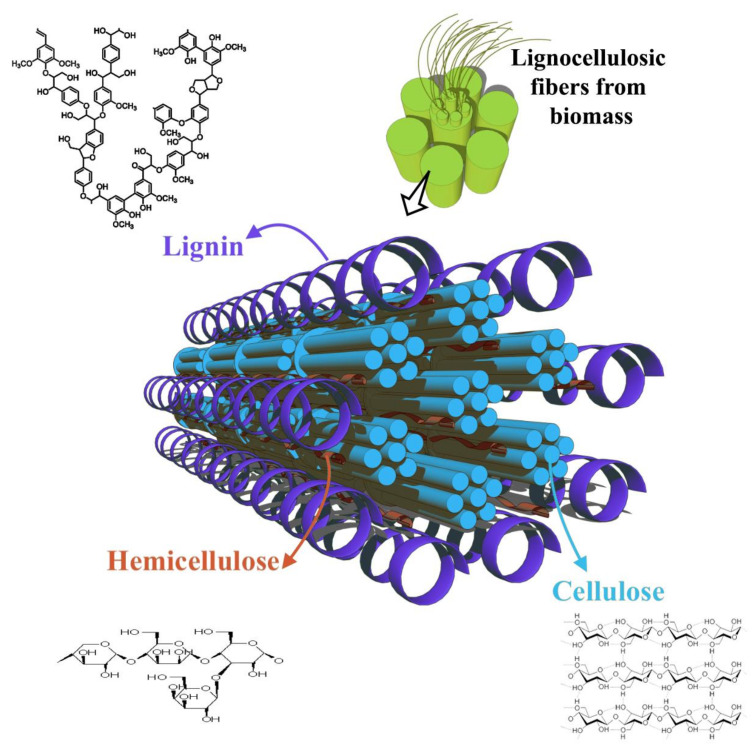
Structure and composition of lignocellulosic biomass.

**Figure 2:**
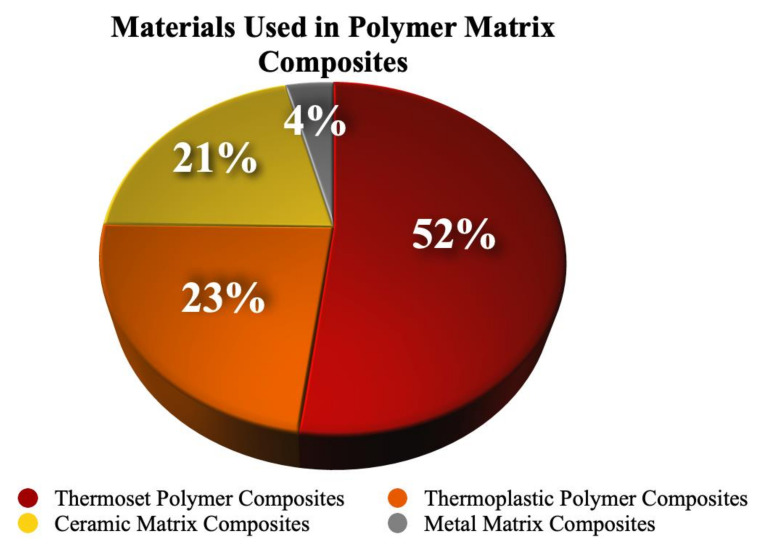
Statistics of materials used in polymer matrix composites globally [24,25].

During carbonization and pyrolysis, biomass is thermally decomposed at different heating rates and residence times. The carbonization process involves longer residence times (24 h) and lower heating rates (1–9 °C/min) [9]. Pyrolysis is a thermal decomposition reaction that occurs in the complete absence of oxygen and yields products such as solid char, liquid, and gaseous hydrocarbon fuels [10,11]. The slow pyrolysis process produces between 15 and 40 wt.% [9,12,13]. The carbonization process occurs between 250 °C and 350 °C and enables the production of a higher product yield of up to 60 wt.% [12,13,14,15]. The most influential factors for biochar quality are the type of biomass feedstock, lignocellulosic composition, and the operating conditions of pyrolysis. The temperature profile and heating rate of pyrolysis affect the physicochemical properties of biochar, including its porosity, pore size, surface area, and tensile strength. These operating conditions also affect the stiffness and water retention capacity of biochar [16,17]. The physical structure of biochar is presented in Figure 1 [14,15,16,17,18,19]. Modifying the structure of biochar greatly affects the filler properties in polymer composites. Its low molecular density makes it a sustainable replacement for various inorganic fillers in polymer composites, such as glass, silica, and silicon dioxide. With calibration, additional properties such as electrical conductivity can also be introduced [19]. The electrical conductivity of biochar increases with higher pyrolysis operating temperatures due to changes in its microstructure [10,20,21,22,23]. Biochar can be used as a filler material in thermosets, thermoplastic, and ceramic polymer composites to improve their mechanical, thermal, and electrical properties. Figure 2 shows the statistics of different fillers which are currently being used in polymer composites.

The shape of fillers affects the filler weight content which typically falls between 25 wt.% and 60 wt.%, while composites with round particles have the highest filler contents [7,15]. Polymer composites with round filler particles achieve higher filler volumes of up to 60 wt.%, while other filler shapes have lower volumes (25–50% by filler volume) [19,24].

The addition of reinforcing fillers in polymer composites improves polymer processing, increases stiffness, and raises the heat distortion temperature, which measures the polymer’s resistance to alteration, and improves creep resistance; additionally, it improves the abrasion, tear strength, and flame retardancy characteristics [2,7,15]. Enforcement of biochar in polymer composites has shown improvement in tensile strength, creep resistance, an improvement on anti-stress relaxation in HDPE by enforcement of biochar at high loads between 50 wt.% and 60 wt.% added composites, as well as maintaining good dispersion and good mechanical structure [12,25,26]. The enforced polymer composite achieved flexural strength of more than 30 MPa, a flexural modulus of up to 1.80 GPa, high tensile strength above 25 MPa, and a tensile modulus above 2 GPa [27,28]. Additionally, higher-surface-area biochar can enhance mechanical interlocking and increase the mechanical stiffness of polymer composites [29].

## 3. Production of Polymer Composites through Extrusion and Casting

Polymer extrusion is a widely used high-volume manufacturing process in which polymer resin, biochar filler, a compatibilizer, and other desired additives are melted and formed continuously. The resulting material is then granulated, fed to a hopper, and processed through a rotating screw in a feed throat [12,14,15]. In contrast, polymer casting involves pouring a liquid polymeric material into a mold with a hollow cavity of the desired shape and size and allowing it to cool to form a solid polymer [12,13,14,15,30]. The polymer extrusion process consists of five stages, beginning with the addition of raw plastic materials like granules and pellets to a hopper, which is then fed into a long heated cylindrical extruder using a rotating screw. The raw material flows down the hopper through the feed throat and large spinning screw within a horizontal barrel. The heating temperature for processing the raw materials is set depending on the type of polymer. The extruded polymer is fed onto a conveyor belt for cooling by air or water medium [31,32,33,34,35]. The polymer die is designed to ensure that the polymer flows smoothly from the extruder and is shaped.

**Figure 3:**
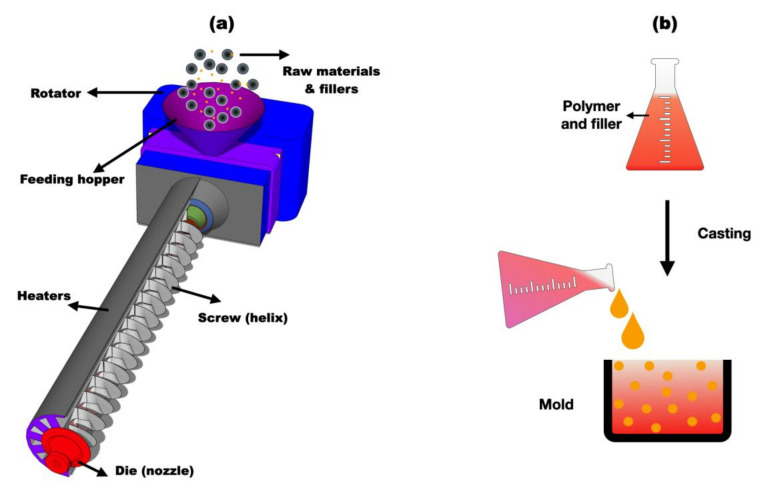
Schematics: (**a**) single-screw extruder with its different parts showing the simplified process of extrusion; (**b**) polymer solution casting method in fabrication of composite materials.

In the polymer casting process, a mandrel or inner diameter mold is immersed in a polymer solution or molten plastic, forming a thin film around the mold. The mold is then extracted in a controlled manner, followed by a curing or drying process [36,37,38,39,40,41,42]. In polymer extrusion, extensional flow orientates the polymer molecules more effectively than in casting. The extruded polymer tends to have fewer inclusions and defects than casted polymer composites due to higher concentration of solid particles in polymer composites [43,44,45,46,47]. The casting process involves introducing liquefied plastic onto a mold and allowing it to cool down and solidify at atmospheric pressure [36,48]. However, polymers with inconsistent viscosity at elevated temperatures, such as polyoxymethylene (POM), polycarbonates (PCs), and polypropylene (PP) are not suitable for the casting process. During casting, the transition from liquid to solid is achieved either by evaporation, chemical action, cooling, or an external heating source [36]. Both described methods are widely used in production of biochar-reinforced composites and nanocomposites. Figure 3 describes the schematic of two main methods in fabrication of nanocomposites in academic and industrial settings.

## 4. Types of Carbon-Based Filler Materials and Their Properties

Utilization of carbon-based composite fillers depends on various factors such as manufacturing cost, tensile strength, and mechanical and electrical properties. Some widely known composites include carbon filaments, carbon nanotubes, and carbon black [7,8]. The addition of carbonaceous fillers enhances heat resistance, stiffness, and creep reduction; reduces flammability; increases compressive strength; improves abrasion resistance; decreases permeability; and improves tensile, impact, and flex strength. It also improves the thermal conductivity, processability, moisture resistance, and adhesion properties of polymer composites.

### 4.1. Carbon Black

**Figure 4:**
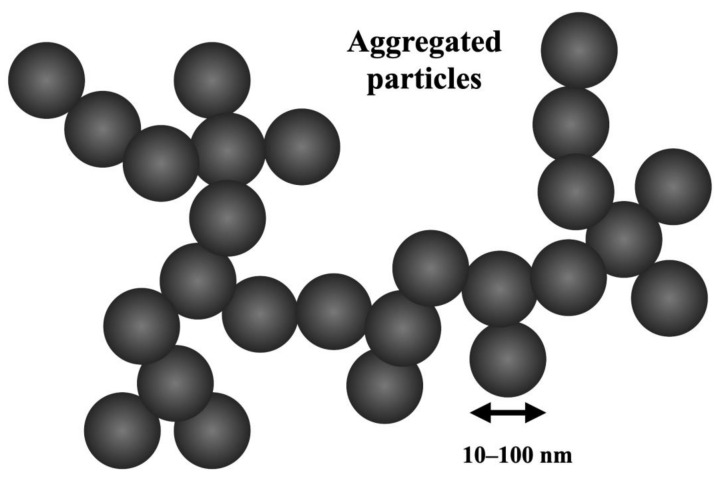
Aggregated particle size of carbon black.

Carbon black is an inorganic material produced by the reaction of hydrocarbon fuels in a limited oxygen supply at elevated temperatures above 1300 °C. Carbon black is a colorant and reinforcing filler used in various products, such as rubber, polymers, and tires, and improves mechanical properties, affects compound properties, such as viscosity, and has a strengthening effect on compounds [7,49]. Carbon black has an amorphous structure and is synthesized through incomplete combustion of coal, tar, or petroleum products, including fuel oil, in the absence of oxygen [50]. Carbon black exists in three forms: thermal black, channel black, and lamp black following different ASTM standards including N220 and N326. Carbon black has the advantage of tunable electrical conductivity in polymer composites. The high environmental impact of the production of carbon black relies on the oil industry as well as environmental and health concerns which is a major drawback for its usage in polymer composites [51]. Carbon black is supplied in various solid forms including powder form and has high melting and boiling point and is derived from petroleum products. It has a specific gravity between 1.8 and 2.2 and is highly combustible with oxidizers. It is a highly sought-after material, whose demand exceeds 8 million tons per annum, primarily used in polymer composites to increase modulus and tensile strength. It is crucial to add an appropriate concentration of carbon black to achieve optimal strength in these composites, as high concentrations may reduce tensile strength at high loadings. The addition of carbon black at the right loading levels is necessary to enhance the mechanical and electrical properties of rubber and polymer composites. It contributes to increase the glass temperature (T_g_) of polymer composites as well as the microhardness [49,51]. The reinforcement effects are controlled through the molecular interaction between polymer molecular structure and carbon black. Experimental investigations found that carbon black increases tensile ductility by a factor of 3 achieving up to 100 MPa in polymer composites, with 7 wt.% filler weight percentage [50]. The addition of carbon black improves the tensile and flexural strength, Young’s modulus, and crystallization behavior of polymer composites by offering more nucleation crystallization sites [52,53]. X-ray diffraction analysis (XRD) identifies the improvements of crystalline behavior using fillers and has shown that carbon black improves the mechanical properties of polymers such as vinyl ester by the addition of carbon black by 5 wt.% [49,51]. Carbon black also prevents ultraviolet degradation and improves plastic conductivity and overall mechanical performance by enhancing abrasion resistance, modulus, tear, and tensile strength [50,54]. Carbon black dispersion and wt.% loading have an impact on the mechanical properties of composite materials which varies between 5 wt.% and 30 wt.% [50,54]. Experimental investigations found that the addition of carbon black by 4 wt.% to ester-based polymer compounds increased tensile strength by 30%, flexural strength by more than 40%, and flexural modulus by over 65%. The optimum Young’s modulus achieved in polymer composites using carbon black was reported at 4 wt.% [51]. Also, the highest improvements in mechanical properties are reported by the addition of polymer composites with less than 10 wt.% carbon black as a filler [54]. The electrical conductivity of carbon black exceeds that of carbon nanotubes at the same volume due to the dispersion limitations and low conduction threshold compared to carbon black polymer composites [55]. The molecular structure and aggregated particle size of carbon nanotubes are shown in Figure 4.

### 4.2. Carbon Nanotubes

**Figure 5:**
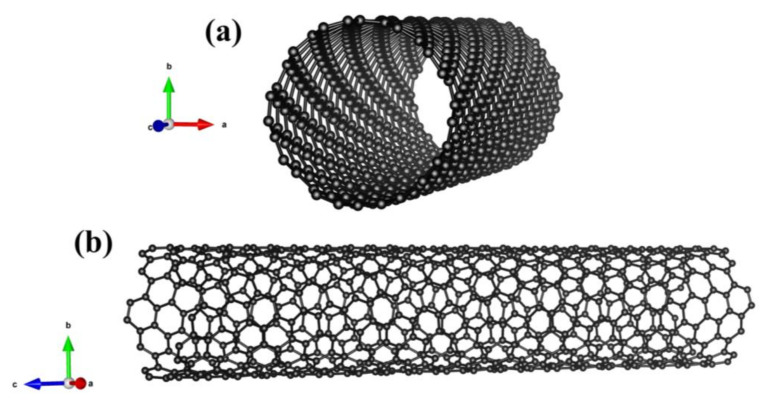
Chemical structure and morphology of carbon nanotubes. (**a**) Cross-sectional view, and (**b**) side view of its structure and atomic arrangement.

Carbon nanotubes are produced from processes such as arc discharge, chemical vapor deposition, and laser synthesis. They are considered a highly effective filler which can potentially improve the performance of polymer composites, enhance the electrical and thermal conductivities, increase mechanical strength, and possess a high length-to-diameter ratio (1000:1). The expected filler concentrations of carbon nanotubes are between 0.5 wt.% and 30 wt.% based on the mechanical structure of the polymer composite [56]. Carbon nanotubes enhanced the mechanical properties and improved Young’s modulus of polymer composites to above 3000 MPa, achieving tensile strength above 30 MPa, and fracture strength that exceeds 100 MPa with more than 3 wt.% filler loading. In rubber composites, modulus and tensile strength have shown improvement by 28% and 25%, respectively, by the addition of 0.1 MWCNT parts per hundred of rubber (phr) [57]. The addition of 3 wt.% MWCNTs increased the toughness and retained the stiffness of polymer composites [58]. CNTs with lower loading (1.75 wt.% by weight) have shown improvement in fracture strength and an increase in thermal conductivity resulting in a reduced number of adiabatic shear bands and a reduction in thermal softening at high cutting velocities. CNTs with high loads above 5 wt.% by weight, affect the mechanical properties and machining response in polymer composites. Also, the addition of carbon nanotubes to ABS composites has increased the flexural module by 17–30% [54]. The high production cost of carbon nanotubes makes them unsustainable fillers in polymer composites. At different CNT loads below 1 wt.%, a 22% improvement in tensile strength is observed in polymer composites while a high flexural strength reaching 400 MPa is achieved by only 0.25 wt.% [59]. Polymer composites with only 1 wt.% CNT have shown improvement in damping properties and better molecular interaction between fiber and polymer composites [60]. The application of carbon nanotubes as a reinforcing filler in polymers enhances electromagnetic interference (EMI) shielding due to its large surface area, 3D networking structure, and unique electronic structure [50,61]. The trends obtained for nanocomposite mechanical properties indicate improvement in storage and loss modulus by increasing CNT loading from 0.5 wt.% to 4 wt.% [62]. The polymer composites containing 4 wt.% MWCNTs have shown similar tensile strength to those with 20 wt.% woody biochar [63]. Mechanical tests on CNTs have shown high elasticity modulus above 1 TPa. Carbon nanotubes have 10–100 times greater mechanical strength when compared to steel at a fraction of the weight. Additionally, CNTs have excellent thermal stability and could withstand up to 2700 °C and electrical conductivity up to 100 S/cm [63,64]. Single-walled CNTs have a hexagonal honeycomb structure of sp^2^ which MWCNTs have multiple concentric tubes circling one another [62]. A major advantage of CNTs in terms of electrical properties is that 4 wt.% loading of MWCNTs indicated the same effect in terms of electrical conductivity as that for 20 wt.% biochar filler in polymer composites. The mechanical structure of carbon nanotubes is shown below in Figure 1. A major disadvantage of carbon nanotubes is their lack of solubility in aqueous media, which hinders their use as a filler material in polymer composites [13]. The molecular structures and aggregated particle sizes of carbon nanotubes are shown in Figure 5.

### 4.3. Carbon Filaments and Fibers

Carbon filaments are composite materials formed by infusing carbon fragment fibers in polymer structures. Examples of widely used filament materials in engineered thermoplastics are polylactic acid (PLA), synthesized nylon polymers, or polyethylene tetra-phthalate glycol (PETG) [12,65]. These are also known as carbon-fiber-reinforced polymers (CFRP), made from a matrix and a reinforcement material. In carbon-fiber-reinforced polymers, carbon filaments improve mechanical strength while polymer resins such as binds improve the polymer matrix and molecular interaction between fillers and polymer composites. It is estimated that carbon fiber is five times more effective as a reinforcement material than steel and has a higher stiffness than steel. In addition, the low weight of carbon fiber makes it an ideal reinforcement material in polymer composites. Carbon fibers synthesized from pyrolysis are added to composites and polymer matrix materials as fillers. Carbon filaments are synthesized using high-temperature catalytic reactions from carbon-based gases at temperatures between 450 and 700 °C [66,67,68]. Carbon fibers are synthesized at operating temperatures above 200 °C by passing oxidized fibers prepared using oxygen and to pass the oxidized fibers at inert conditions and operating temperatures between 400 °C and 1500 °C for fiber carbonization [66]. Integration of carbon fibers into polymer composites such as acrylonitrile butadiene styrene (ABS) increased tensile strength by 39%, reaching 37 MPa, and increased Young’s modulus by 60%, reaching 0.79 GPa [69]. Conventional carbon fibers and filaments produced from biomass pyrolysis differ in the carbon microstructure. To produce highly effective fibers with a high degree of orientation, precursor fibers are given mechanical stretching treatment during the pyrolysis process [70]. Carbon filaments are synthesized by a top–down approach using thermal treatment techniques formed by infusing fragments of carbon fibers in polymers. Carbon filaments are produced in a rotating quartz tube furnace under inert conditions at operating temperatures between 400 °C and 1600 °C. The precursor fiber could be either polyacrylonitrile or pitch fiber precursor through heated oxygen where oxygen is absorbed and then passes through the carbonization process. Carbon filaments are then synthesized by infusing fragments of carbon fibers at operating temperatures between 250 °C and 700 °C in a batch process [69,70,71]. High carbide content is required as a prerequisite for the nucleation of carbon filaments. Table 1 presents a summary of the mechanical properties, including elastic modulus, tensile modulus, and ultimate strain, of different carbon-based fibers.

Carbon fiber layers consist of carbon filaments that have carbon layers whereas the filament arrangement is affected by the synthesis process conditions during the filament fabrication [28]. At the same volume fraction, carbon filaments have lower tensile strength and stress compared to carbon fibers with the same volume in a reinforced polymer composite [68]. For instance, carbon filaments at a filler loading of 18 vol.% have 20 times higher tensile strength compared to the same volume fraction of carbon fibers yielding a tensile strength of more than 20 MPa [67,68]. Table 2 presents a comparison between biochar, carbon nanotubes, and carbon filaments as fillers.

### 4.4. Graphite and Graphene-Related Materials

Graphene, a single layer of graphite, possesses distinctive characteristics such as remarkable conductivity, strength, and thermal stability. These exceptional properties make it possible to integrate this material into polymer composites as nanofillers, thereby improving their overall properties. The trend in recent years has seen a shift towards slimmer and more compact designs for electrical devices. Consequently, effective heat management has emerged as a crucial consideration in both device and application design. This challenge extends to various other applications like electric motors, generators, power generation heat exchangers, and automotive systems, all of which grapple with similar thermal issues. The incorporation of thermally conductive fillers into polymer-based composites has significantly enhanced their thermal conductivity performance. As evident from the aforementioned points, graphite is gaining increasing popularity for its ability to improve the thermal conductivity of composites across a wide range of applications. The nanostructure of graphene allows it to be an effective sustainable filler for different kinds of thermoplastic polymers or thermosetting resins with unique mechanical and chemical properties.

In the presented study, the solution-casting method was employed to fabricate PSU-based composites filled with various filler concentrations, reaching up to 70 wt.%. Three different types of graphite were utilized as fillers to enhance both thermal conductivity and mechanical properties. Upon investigating the morphology and structure of these fillers, it was observed that natural graphite possessed a flawless crystalline graphite structure, making it the most effective filler in enhancing the composite’s thermal conductivity [74].

Regarding the thermal conductivity of the composite, the highest value was achieved using 70 wt.% natural graphite, reaching approximately 4.26 W m^−1^ K^−1^. This superior performance can be attributed to natural graphite’s perfect crystalline structure and its large aspect ratio, which results in a substantial conducting surface area interacting effectively with the matrix, facilitating more efficient heat transfer compared to other types of graphite [75].

### 4.5. Biochar

Biochar is a carbon-rich material, which is abundant and prepared easily from various types of biomasses. Biochar has superior characteristics including hydrophobicity, high surface area, and porosity, which improves its mechanical and thermal properties [2,23]. Addition of biochar improves the thermal stability of polymer composites due to the nitrogen- and oxygen-containing functional groups existing on the surface of biochar [4,69]. It also improves the physical adsorption capacity of polymer composites through enhanced molecular interactions such as hydrogen bonding [2,22,76]. Additionally, the thermal stability of biochar can be adjusted between 0.10 and 0.13 W m^−1^ K^−1^ depending on the different pyrolysis temperature profiles which produce differences in microstructure, minerology, and physicochemical properties [4,22,76,77].

The incorporation of biochar- or wood-based materials into the matrix increases the polymer hardness by achieving mechanical interlocking of the polymers with biochar; this is possible because of the enhanced matrix wettability granted by the large surface area of biochar. The bulk properties of polymer composites are predicted using various theoretical models such as the Halpin–Tsai–Nielsen and Verbeek models. Addition of biochar increased different polymer composite hardness.

By increasing the operating temperature from 600 °C to 800 °C, the porosity and surface area increase, forming graphite-like molecular domains which contribute to increase in the mechanical hardness. Higher operating temperatures lead to progressive enlargement and ordering processes of graphic domains through turbostratic rearrangement [2,69,70]. The properties of biochar, such as surface area, porosity, and grindability, are controlled by several factors, such as d morphology, chemical composition of feedstock, and the type of post-treatment technique used, e.g., physical activation or surface tailoring [70,71,78]. The thermochemical processes chosen for production of biochar play a critical role in determining the final physical properties of the biochar filler. The different parameters such as production temperature, reactor dimensions, and lignin content in feedstock play a role in controlling the porosity and surface area of biochar [70,78].

A decrease in the pyrolysis operating temperature to 450 °C causes a decrease in the functional groups and reduces the affinity of biochar towards polar moieties [79,80]. Biochar produced at high temperatures above 700 °C has decreased O/C and N/C ratios due to temperature-induced dehydration and the carboxylation process [4,81,82]. Additionally, the solvent absorption capability of the biochar improves due to the formation of more non-porous structures. The development of fire resistance in biochar is a result of the formation of compact carbonaceous layers which prevent the transfer of oxygen towards the polymer matrix [4,81].

The mechanical characterization of biochar polypropylene composites show that the addition of between 20 and 24 wt.% of biochar produces similar tensile strength but higher modulus strength and flexural properties [4,81]. However, the addition of biochar beyond 15 wt.% in polymer composites reduces the material ductility. In terms of fire-resistant properties, biochar samples produced from pyrolysis at 500 °C and activated at 900 °C have shown exceptional fire-retardant properties [83].

The type of biomass feedstock influences the main mechanical characteristics of biochar such as pore volume and ash content. Biochar with higher carbon content and higher surface area exhibited higher tensile strength and modulus [83]. Biochar with high CaCO_3_ loadings have shown enhanced fire-resistant properties due to the inorganic content hindering the diffusion path of oxygen in polymer composites [4,82,84]. Furthermore, increasing biochar content decreases the heat release rate and smoke production rate [4,77]. Introduction of higher biochar loadings also causes a monotonic increase in the tensile and flexural moduli due to peculiar morphology of polymer composites [2,22,77].

The morphological properties of biochar as well as mechanical interlocks make it an excellent filler in various polymer composites. The enhanced matrix wettability of biochar caused by high surface area improves the mechanical interlocking properties of polymer composites [4,77,85]. The moisture-holding capacity of biochar can be altered by changing the operating temperature of pyrolysis or carbonization, which further improves the moisture-holding capacity in polymer composites [4,22,77]. The existence of turbostratically ordered regions, arranged crystalline regions, and disordered amorphous regions in cellulose enhance the electrical conductivity of biochar [2,22,70,78]. The honeycomb-like pores on the biochar surface results in the improved mechanical interlocking of the polymer with biochar causing it to be an excellent reinforcing agent [70,78,86,87]. The highest improvement in tensile strength in polymer composites was observed by the addition of 15 wt.% biochar resulting in a higher stress yield. Polymer composites with higher biochar-loading rates (25, 30, and 35 wt.%) have shown semi-brittle behavior under tensile strength [17,22,70,77]. The addition of biochar also increases the tensile modulus. Biochar loading at 35 wt.% has shown a tensile modulus of higher than 3080 GPa [4,77,85]. Biochar produced by carbonization or pyrolysis at high operating temperatures of 900 °C enables better stress transfer between polymer composite and biochar [4,22,77,85]. The addition of biochar also increases stiffness, resulting in a lower percentage elongation for polymer composites, with the best elongation reported at 15 wt.% biochar concentrations [88]. Both the flexural strength and modulus have shown improvement with addition of biochar at high loadings [2,69,70].

Also, the stress transfer caused by the addition of biochar in polymer composites improves the mechanical properties between the filler and the matrix by obstructing mechanical failure. The addition of biochar improves the cross-linking of the epoxy matrix by blocking the movement of molecules, which in turn increases the strength of the polymer composite [22,78,79,89]. The addition of biochar also enhances thermal conductivity values and improves the tolerance of strain before reaching the glass transition temperature (T_g_) [71,78,86,87]. The ultimate strength of around 40 MPa for polymer composites is achieved by lower biochar filler loading; the strength decreases with an increase in the biochar-loading rate. Deterioration in the load bearing capacity of polymer composites have been shown to be a result of the transition of the polymer composite behavior matrix from plastic to semi-brittle at high biochar load-bearing capacities [21,78,86]. The impact strength for polymer composites is improved by the micro-hardness features of biochar [79,89]. Biochar filler particles with softer phase of hardening agent increase the degree of stress transfer to filler particles [78,86]. The temperature of biochar carbonization affects the mechanical properties and the tensile modulus of polymer composites.

## 5. Use of Filler Materials in Polymer Composites: Effects and Limitations

Many polymer composites are prone to decomposition at high temperatures, which can limit their applications in certain industries and environments. Moreover, polymer composites, especially those reinforced with fibers, can be highly sensitive to moisture absorption. The other limitations of polymer composites include poor toughness, bending strength, and creep performance, which is defined as the tendency of the material to deform permanently under influence of mechanical stress [60,66,90]. The polymers owing to these characteristics can undergo dimensional changes via either thermal or mechanical degradation and hence decrease the strength and stiffness properties. Additionally, the high fabrication cost of polymer composites at low production rates limits the widespread adoption of polymer composites in various industries; therefore, new manufacturing technologies are required to reduce fabrication costs and make polymer composites more economically viable [63,68]. Emerging technologies such as the incorporation of organic sustainable fillers, e.g., biochar, aim to enhance the physical performances of polymers by increasing their tensile strength and melting temperatures. However, the tensile strength of the polymer matrix can be improved by the addition of compatibilizers, which are the coupling agents added to immiscible materials during the extrusion process. This alters the interfacial properties, stabilizes the melt blend [78,91], and enhances the mechanical bonding strength and cross-linking of immiscible materials, such as biochar and polymer materials, as well as enhancing the interfacial adhesion between polymer composites and compatibilizers [90]. Non-reactive compatibilizers, including ethylene–ethyl acrylate (EEA), are used as polypropylene polymers. Other compatibilizers, such as ethylene–butyl acrylate and ethylene methacrylate (EMA) copolymer, used for the compatibilization of polypropylene, polyethylene, polyethylene terephthalate, and acrylonitrile butadiene styrene, are most widely used in polymer composites. However, frequently used reactive compatibilizers include acrylic-based/grafted polyolefin polymers, polyethylene, polypropylene, and polybutylene terephthalate. The addition of compatibilizers to various polystyrene and polypropylene compounds improves their mechanical properties, increases their tensile strength by 20%, and increases their impact strength by 100% [60].

Various carbon-based fillers, including carbon black, are the oldest carbon fillers employed in the plastic and rubber industries. However, carbon fiber and graphite are more versatile carbon alternatives because of their low cost and their capacity for mass production, placing them at an advantage among other carbon-based polymer composites [92]. The enhancement of the physical properties of polymer composites by the addition of fillers depends upon several structural parameters: homogeneous composition, morphology, size, and aspect ratio of the fillers; the surface characteristics; the surface area; the interfacial filler–filler interactions; and polymeric material and filler, etc. Additionally, the processing and operating conditions, such as the mixing technique, loading rate, shear effects, temperature, etc., have an observed effect [92]. Several researchers have focused on the mechanical, thermal, and electrical characteristics of carbon-added polymeric composites to investigate their potential applications in advanced technologies, including batteries, sensors, solar cells, bipolar plates, and thin film applications [73,93,94]. For instance, the addition of biochar has shown to bring about an increase in the oxidative stability and thermal decomposition temperature (by 15 °C) of polymer composites [29,90]. However, the addition of organic fillers to polymeric materials leads to distinct characteristics compared to control polymeric materials; this requires further investigation to be better understood.

### 5.1. Effects of Filler Addition on the Mechanical Performance of Polymer Composites

There are several mechanical limitations of polymer composites that could be improved by biochar organic fillers such as lower fracture toughness, flexural strength flexural modulus, and hardness [73,78,91]. As the volume of filler increases, the modulus of rupture and the flexural modulus, as well as material toughness, increase. The most recommended filler volume for mechanical toughness is approximately 55% by volume [90,94]. The shape of biochar filler particles influences filler volume. For example, irregular biochar fillers have low filler volume (between 25 wt.% and 51 wt.%), whereas biochar in round particles has high filler content up to 60 wt.%. Polymer composites with high filler content exhibit high flexural strength up to 120 MPa, flexural modulus (12–15 MPa), and the highest toughness achieved at filler volume 55 wt.% [94].

### 5.2. Thermal Conductivity in Polymer Composites

**Figure 6:**
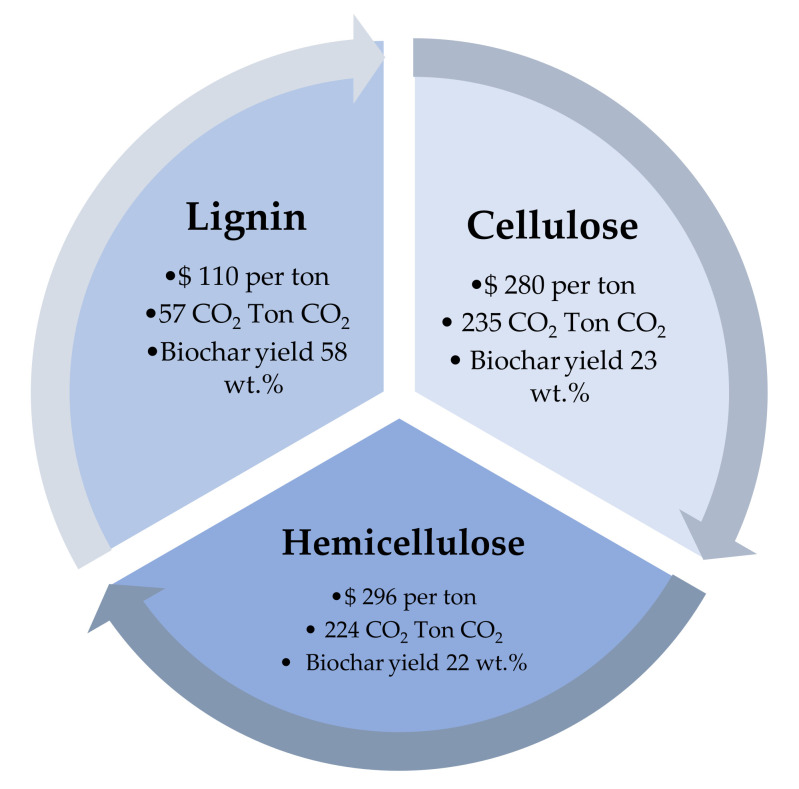
Slow pyrolysis: expected costs and CO_2_ emissions from different components of feedstocks from slow pyrolysis [15]. This provides examples of various interactions on the surface of biochar particles and biochar–matrix interactions.

Thermally conductive polymer composites are considered to be promising materials due to their multiple applications in electronic equipment [95]. Biochar fillers in polymer composites achieve good tensile and flexural properties and improve the thermal stability and ductility of polypropylene-reinforced composites [96]. The addition of biochar enhances thermal properties due to the high surface area and microhardness of biochar that achieves mechanical and thermal improvements in various polymer composites. One of the current limitations of reinforced polymer composites is their low electrical conductivity compared to metallic-based fillers, which limits their industrial applications [97]. The composition of the feedstock also affects the expected cost and CO_2_ emissions from slow pyrolysis. Cellulose, hemicellulose, and lignin are the main components of most biomass feedstocks, and they have different properties and behaviors during pyrolysis. For example, cellulose is the most reactive component and tends to produce more gases and char, while lignin is more stable and tends to produce more solid and liquid products. In general, lignocellulosic biomaterials (such as wood and agricultural residues) represent a more cost-effective type of feedstock for slow pyrolysis in the production of char, which can be used as a filler material in polymer composites. Figure 6 presents the expected biochar product yields and CO_2_ emission rates from different types of feedstocks.

## 6. Biochar Properties and Biochar’s Impact as a Filler in Polymer Composites

Slow pyrolysis at lower heating rates (<10 °C min^−1^) produces a high biochar product yield and is recommended due to the high conversion rate and ease of operations, economic feasibility, and maintenance [98,99]. Biochar physicochemical properties are regulated by feedstock properties and pyrolysis conditions, such as temperature profile, reaction time, reactor configuration, and temperature profile [98,100,101]. During carbonization, heating rate and heat dissipation affect the physicochemical properties of biochar which influences the energy content, product yield, carbon content, porous structure, and biochar pH value. At high operating temperatures, the pore size and volume increase, and the increase in the biochar surface area is caused by the change of carbon phase from an amorphous structure to graphite. For example, the biochar surface area at 400 °C from sawdust has a surface area of 147 m^2^ g^−1^ and increases to 570 m^2^ g^−1^ by increasing the operating temperature to 700 °C [2,4,70,89]. It is necessary to develop methods which can produce high biochar porosity and longer pyrolysis residence time that exceeds 60 min [102]. Physical activation methods are carried out using carbon dioxide or steam, while chemical treatment activation is carried out by acid, alkaline, and oxidizing or reducing agents to increase biochar surface functionality. Biochar properties, including surface area, pore size, and pore structure, increase adsorption capacity and carbon dioxide storage capacity; this improves both surface functionalities and mechanical properties [103,104]. Biochar’s inherited properties are greatly dependent on its physical structure and operating conditions during biomass pyrolysis. Properties such as electrical conductivity are also controlled by the internal structure of biochar [2,4]. Slow pyrolysis is the most frequently recommended thermochemical reaction for producing a large yield of biochar. Depending on the biomass feedstock used, e.g., wood, straw, green waste, or dry algae, biochar yields vary from 20 wt.% to 90 wt.%, depending on operating conditions [70,98,105]. It is noted that, at higher pyrolysis operating temperatures, the highest biochar yields are achieved. For example, for slow pyrolysis using wood biomass, at 300 °C and 10 min residence time, biochar yield is 89 wt.%, while at 600 °C and 10 min residence time, biochar yield is higher [68,105,106]. Table 3 presents a summary of biochar and hydrocarbon product yields obtained from different pyrolysis and carbonization operating conditions [1,9,107].

Rotary kiln reactors are recommended for slow pyrolysis and high-temperature reactions with long residence time at slower heating rates for increasing biochar product yield. However, this type of reactor is not recommended for high-level production of bio-oil. More specialized reactors have been developed that produce higher biochar yields [70,98,105]. The most important operational factors in chemical reactors are the operating temperature, the heating rate, the separation and cooling of vapors, and the gas cooling system. In fixed-bed reactors, solid residue and biochar are collected at the bottom of the reactor and contacted by either a counter or a co-current gas stream. Biochar is collected from the bottom of the chemical reactor. Fixed-bed reactors are mostly recommended for slow pyrolysis and carbonization processes which have longer residence time. Fixed-bed reactors could be used in biomass carbonization, pyrolysis, or gasification [100]. Fluidized-bed reactors are mostly recommended for biomass gasification reactions. Fluidized-bed reactors are recommended for higher heat and mass transfer between solid and gas particles. Fluidized-bed reactors are efficient reactors for rapid heat transfer with different configurations including vertically upward and vertically downstream. Fluidized-bed reactors are either heated by direct heat or indirect heat transfer. Another advantage of fluidized-bed reactors is their ability to operate at lower operating temperatures for a long residence time. The optimal biochar yields produced from biomass in fluidized-bed reactors are between 25 wt.% and 45 wt.%; these are affected by the heating rate and the temperature profile used, depending on the operating conditions and lignin content in biomass feedstock [2,71,89]. Ablative reactors are recommended for fast pyrolysis for processing various types of lignocellulosic feedstock with high energy density requirements. Ablative reactors are mostly recommended for more intense reactions without the need for inert gas to operate. Ablative reactors are recommended for intense reactions with high heat transfer. An advantage of ablative reactors includes a high recovery rate of liquid and gaseous products under high pressure caused by mechanical centrifugal force. Pyrolysis reactions are carried out in ablative reactors between 500 °C and 600 °C [3,70,78]. Augur reactors have a similar product yield for hydrocarbon liquid products and char, like those from fluidized-bed reactors. Augur reactors have higher heat transfer efficiency and yield high char production from slow pyrolysis. For fast pyrolysis, liquid hydrocarbon product yields are maximized. Biomass is rapidly converted to bio-oil, biogas, and biochar. Biochar drops to the bottom of the reactor and hydrocarbon gases and liquids collect into the cyclone. Spouted-bed reactors are recommended for biomass flash pyrolysis that allows continuous feeding and versatility in gas flow; this makes this reactor suitable for handling solid particles, such as biochar. It produces high yields of biochar compared to other reactor configurations.

Different kinds of composite materials are based on the polymer matrix and can develop high-performance polymers with better mechanical, thermal, and chemical resistance that can withstand harsh environments and applications with a reduction in production costs [64]. Carbon-based materials, such as carbon black, carbon nanotubes, graphene, and carbon filaments are introduced into polymer composites due to ease of use, large surface area, and high thermal, chemical, and mechanical stability, and can be used as nanofillers to enhance the performance of polymeric membranes [13,59]. Biochar is considered a cheap biomaterial, and its physical properties could be controlled via operating conditions of pyrolysis or carbonization. Biochar’s high surface area and high mechanical performance have shown up to 35% improvement in flexural strength compared to other filler materials such as conventional glass-fiber-reinforced polymers (GFRPs) [29]. Additionally, the addition of biochar to polymer composites could reduce their material density. At higher temperatures of pyrolysis, biochar’s pore volume and surface area increase due to lower biochar particle density [109,110,111]. The tensile performance of polymer composites is tested according to ASTM-D 638 standards. The addition of 4 wt.% MWCNTs achieves a 13% higher Young’s modulus, while the addition of 20 wt.% as an organic filler increases the Young’s modulus of polymer composites by 60% [63]. Therefore, biochar is considered to be a better and cheaper filler in polymer composites related to CNTs. Also, the integration of biochar in polymer composites increases the hot–wet use temperature and fracture toughness of materials that could be used in aerospace applications. Biomass feedstock also has an impact on the quality of biochar and filler properties in polymer composites. Factors that affect biochar’s properties are the pyrolysis operating temperature, the reactor design, and the feedstock; these control the physical performance of biochar, e.g., the particle size and porosity [112].

Biochar with high porosity achieves higher thermal stability in polymer composites. Biochar is compared to various organic and inorganic fillers. This section summarizes the effects of biochar properties and pyrolysis conditions on polymer properties in terms of crystallinity, thermal stability, flammability, electrical conductivity, and improvement in mechanical and electrical properties. Biochar has an impact on the crystallization temperature (T_c_) and thermal performance of polymer composites [105]. The higher surface area in biochar, lower bulk density, high cation exchange capacity (CEC), and high carbon content have a positive impact on the mechanical and electrical performance of biochar in polymer composites [4,106]. The degree of crystallization is measured using an X-ray diffractometer to analyze the crystalline structure of the reinforced polymer composite.

Biochar polymer aspect ratio, biochar filler loading, and nucleation have an impact on the crystallization properties of polymer composites. The biochar surface area and microporous structure is controlled by pyrolysis operating conditions and act as a nucleation agent for crystallization. The addition of biochar particles into a polymer composite structure improves the overall thermal performance and shifts the crystallization temperature (Tc) ten times higher in polymer composites [105]. A thermogram of polypropylene-reinforced polymers shows that biochar raises their crystallization temperature and melting enthalpy. The degree of crystallinity (X_c_) after biochar enforcement in polymer composites is calculated using Equation (1):

(1) $$X_{c}\left(\%\right)=\frac{∆H_{m}}{\left(1-\emptyset \right)∆H_{m}{}^{\circ}}$$

where X_c_ is the crystallinity in biochar, ∅ is the biochar weight fraction in the polymer composite, and $∆H_{m}{}^{\circ}$ is the enthalpy of melting a 100% crystalline neat polymer composite. The thermogravimetric analysis can be used to measure the thermal stability of biochar in polymer composites, while polarized light microscopy can be used to investigate the impact of the addition of biochar on the morphological behavior of biochar in polymer composites [105].

The thermogravimetric analyzer calculates the thermal stability of biochar in polymer composites, while polarized light microscopy is used to observe the effect of biochar incorporation on the morphology of biochar and polymer composites. Results have shown that the incorporation of biochar in polymer composites increases the thermal stability and thermal resistance of polymer composites; allowing them to tolerate temperatures 80 °C higher than those tolerated by neat polymer samples [105]. Biochar has shown similar electrical performance compared to graphene and carbon black with better environmental aspects such as recyclability and sustainability. More experimental analysis is needed to determine the optimal biochar loads and the effects of the physical properties of biochar to optimize the mechanical and electrical performance of polymer composites using cost-effective manufacturing techniques.

### 6.1. Effects of Biochar Addition on the Thermal Stability of Polymer Composites

Enforcement of biochar as a filler has a positive impact on the thermal properties of polymer composites and improves the thermal performance, as well as increasing the melting point and the flammability limit of polymer composites. Polymer composites with biochar fillers have shown higher thermal stability than neat polymers. For example, the addition of 15 wt.% biochar to polypropylene increases the degradation temperature from 390 °C to 412 °C. Thermogravimetric analysis can be used to measure the thermal stability of polymer composites with different biochar loads; for example, biochar at 20 wt.% loading was found to increase the thermal stability of polypropylene to temperatures which were 80 °C higher than those tolerated by samples without biochar [105]. Biochar at certain loads causes a significant decrease in thermal conductivity and diffusivity [77,84,86,112,113,114]. Experimental results have shown that the addition of 5 wt.% of biochar increases the tensile modulus and improves the mechanical strength of various polymer composites [115]. Hence, the usage of optimized biochar loading improves the mechanical strength of plastic composites as well as their thermal stability. Addition of higher loads of biochar decreases the hydrophobic behavior of the polymer due to the hydrophilic characteristics of biochar [115]. Polymers have poor mechanical properties; biochar fillers could enhance the mechanical, thermal, and electrical properties of polymer composites. The high surface area of biochar improves the mechanical interlocking and thermal stability of polymer composites, which can be validated using thermogravimetry [78].

### 6.2. Effects of Biochar Addition on Flammability and Flame Retardancy

The thermal stability of polymer composites, such as polypropylene, has been shown to be increased by biochar loadings of 15 wt.%, 25 wt.%, 30 wt.%, and 35 wt.%; these can be processed using different mechanical processes, such as injection molding and extrusion [90]. Polymer composite mechanical testing is performed using cone calorimetrics, thermogravimetric analyzers, X-ray diffraction, and IR spectroscopy [89]. Experimental investigations have found that biochar at 10 wt.% loading in polymer composites achieves the highest flame-retardant properties and thermal stabilities [116]. The loads of biochar which are most recommended for enhancing mechanical properties are those between 15 wt.% and 30 wt.%; higher loadings of biochar deteriorate the mechanical properties [90,117,118]. The addition of biochar reduces the peak heat-release rate and smoke-release properties of polymers due to the flame-retardant properties of biochar. Biochar with higher surface areas, produced at high operating temperatures, creates a mechanical engagement that improves the mechanical properties of polymers [119]. High thermal stability, measured using thermogravimetry, has been detected in polypropylene composites, and this is attributed to the flame-retardant properties of biochar as a filler [119]. The addition of biochar has been shown to improve heat resistance, increase resistance to flames, reduce smoke-production rates [120].

### 6.3. Electrical Conductivity of Polymers by Addition of Conductive Fillers

**Figure 7:**
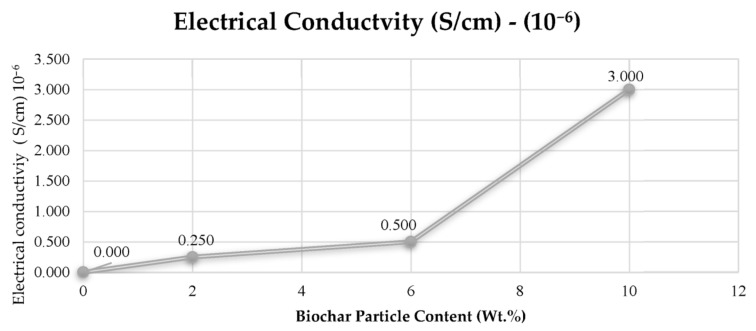
Electrical conductivity at different filler loadings of biochar in PVA polymer composites [22,23].

All polymer composites have a degree of preferred physical orientation in terms of resistivity measurement. For electrically conductive fillers, the mechanical behavior of polymer composites decreases with increasing filler volume fractions [74]. At a constant filler volume, as the carbon fiber length increases, the electrical conductivity decreases due to higher resistivity. For example, at a constant filler volume of 40 wt.%, the resistivity of 200 μm is 0.038 ohms, while the resistivity of 400 μm is 0.011 ohms [74]. The electrical conductivity in polymer composites can be measured using several techniques, such as voltmeters [117,118,121]. Figure 7 presents an example of the measured electrical conductivity in polyvinyl alcohol polymer composites at different biochar loads of 2 wt.%, 6 wt.%, and 10 wt.%.

The usage of fiber-reinforced polymers with continuous filaments of highly electrically conductive materials improves the electrical conductivity through the creation of interconnected pathways that improve current flow [122]. Experimental investigations have shown that the existence of orthogonally conductive z-filaments in carbon epoxy composites creates conductive pathways; this can be used in the development of electrically conductive biochar. There is a demand for the development of highly conductive carbon filaments to improve electrical conductivity in various composite materials, as well as their mechanical and thermal properties [123].

### 6.4. Impact of Biochar Filler Loading on Other Mechanical Properties

The replacement of carbon fibers with carbon filaments in composite materials has shown improvement in electromechanical performance and noise control [67]. Experimental investigations have shown that the usage of short carbon fibers favors high conductivity and polymer properties are affected by filler content and glass temperature as well as the degree of filler dispersion. Also, investigations suggested that energy consumption increased with higher dispersion of filler content [90]. Biochar extracted from poplar plants mixed with HDPE improves the flexural strength of HDPE polymer composites. However, biochar loadings higher than 70 wt.% lead to a reduced flexural strength compared to pristine HDPE [61,86,105]. Higher biochar content leads to a higher agglomeration degree that improves mechanical properties, such as flexural strength, compared to neat polypropylene from 50 MPa to 59 MPa with the addition of more than 30 wt.% biochar [78,90,105]. Polymer composites with high flexural strength reaching 140 MPa had biochar loading at 55 wt.% [96].

**Figure 8:**
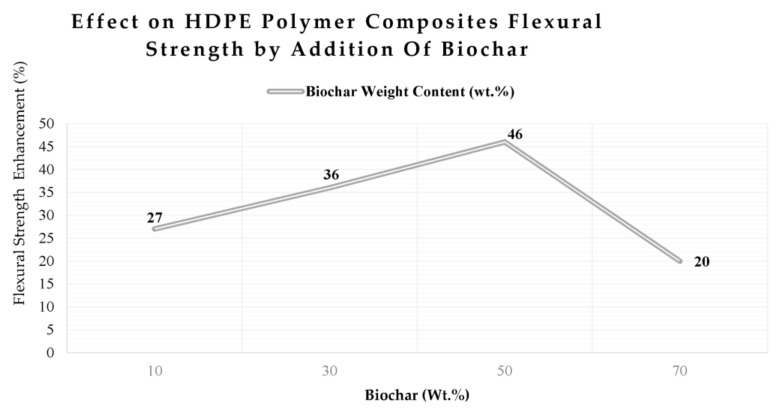
Effects of the addition of different loads of biochar on the flexural strength of HDPE polymer composites [83].

Experimental investigations have shown that biochar obtained at lower pyrolysis temperatures (300 °C to 400 °C) has the highest tensile strength, whereas biochar obtained with pyrolysis temperatures between 600 °C and 900 °C has the same mechanical strength. Biochar derived from bamboo plant shells has shown better mechanical properties than other types of biochar such as coconut shells due to high fiber content [124]. Biochar produced from the bamboo plant has shown better water resistance, thermal stability, and mechanical properties. The optimal temperature for achieving hydrophobicity in polypropylene composite materials is found at 300 °C for biochar [124]. Also, the addition of biochar at different loads to thermoplastics such as polylactic acid (PLA) has different effects based on different biochar (BC) loads ranging from 5 wt.% to 25 wt.% [86,98]. For example, biochar loading at 5 wt.% improves the elasticity tensile modulus by 38% and decreases the tensile strength by 5% [115]. Increasing the biochar content could lead to a reduction in the tensile modulus of elasticity [115]. For example, as shown in Figure 8, the optimal loading of poplar biochar to HDPE composites at 46 wt.% achieves the highest flexural strength while any further addition of biochar weakens the HDPE polymer flexural strength [82]. Table 4 summarizes the improvements in acoustic, thermal–mechanical, and electrical properties of different polymers at various biochar loads.

Polymer composites with 4 wt.% biochar have shown high tensile strength and 180% higher strength compared to neat polymers. Also, at 4 wt.% biochar, significant improvement in thermal resistance is noticed in polymer epoxy composites [14,15]. Also, an experimental trial highlighted the impact of different biochar loads on Young’s modulus of biochar filler in polypropylene polymer composites as shown below in Figure 6. It is concluded that the impact strength shows a massive increase once biochar loading increases more than 25 wt.%. In addition, increasing the biochar filler by 5 wt.% have shown improvement in tensile strength and hardness of polymer composites as well as thermal resistance [111].

**Figure 9:**
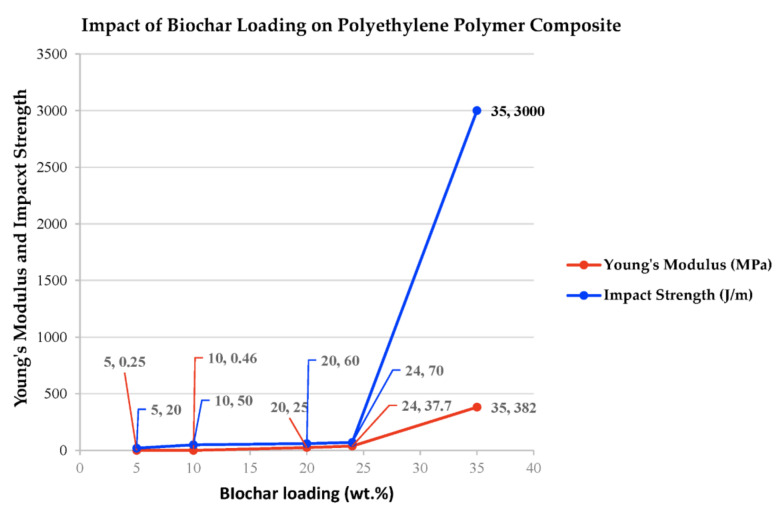
Impact of biochar loads in polypropylene polymer composites in terms of Young’s modulus and impact strength [12,50,137].

As shown in Figure 9, optimum biochar loading in HDPE polymer composites to improve impact strength are between 23 wt.% and 35 wt.% reaching high impact strength of 3000 J/m. Also, experimental investigations have shown that biochar loadings at 25 wt.% in polypropylene polymer composites have the same tensile strength and modulus as similar properties in wood composites but higher flexural properties [16,90].

### 6.5. Effects of Biochar Particle Size and Loading on Polymer Composites

Polymer composites can be processed using extrusion and injection molding methods. Biochar can be incorporated into polymer composites through grinding, milling, and extrusion techniques. The thermal treatment and operating temperature of biochar can affect its porosity and mean particle size distribution, which in turn influences the behavior of the polymer composite [122]. While biochar offers high thermal stability, composite enhancers may be required to increase the mechanical mixing of biochar filler and polymer composite. The mechanical and electrical performance of biochar fillers in polymer composites is also influenced by the particle size and porosity of biochar. Different operating conditions during biomass pyrolysis and carbonization can result in varying mean pore sizes, thermal and electrical conductivity, and mean particle size and distribution [122].

Biochar loading affects the chemical, mechanical, and electrical properties of polymer composites. The addition of biochar enhances thermal properties, such as increasing the crystallization temperature, because the nucleation sites in biochar are available for crystallization [15,116]. The addition of biochar also improves the mechanical performance of polymer composites by transferring stress from the polymer matrix to the biochar filler, increasing mechanical resistance and preventing failure. For instance, the addition of 35 wt.% biochar in polymer composites increased the tensile modulus up to 3.80 GPa [110].

The improvement of mechanical properties of polymer composites is caused by high porosity in biochar, which causes stress transfer between the filler and the polymer matrix, obstructing mechanical failure. Biochar obtained at lower pyrolysis temperatures improves wettability and molecular affinity, lowers the friction coefficient, and enhances the biocompatibility of polymer composites [7,91]. The improvement of thermal conductivity in polymer composites is a result of the faster heat diffusion generated by the biochar and its ability to tolerate higher strain before reaching the glass transition temperature (T_g_). The highest tensile strength achieved by biochar in polymer composites is achieved at lower loadings. In addition, the higher filler concentration and cross-linking of biochar increase the brittle behavior of polymer composites. The addition of 2–4 wt.% of biochar loading in polymer composites modifies the tensile behavior from brittle to ductile. With the addition of 2 wt.% biochar, the tensile strength and elongation increase by 45% and 400%, respectively [90,135]. Biochar in polymer composites improves the viscosity of the composites; additionally, the biochar particle size has massive effects on the flow behavior and leads to a severe increase in shear viscosity to around 5000 Pa-s [122]. Biochar with smaller particle size diameter has shown lower viscosity compared to samples with large particle diameters. At lower loadings, biochar improves the elasticity of polymer composites and increases the stiffness and overall mechanical properties of polymer composites [116]. The addition of optimal concentrations of biochar also improves the flame-retardant properties of composites [138]. Table 5 presents a summary of different preparation methods for biochar-based polymer composites, as well as their limitations and applications.

### 6.6. Effects of Biochar Surface Area

Surface modification of biochar can help remove impurities and improve adhesion with the matrix interface, leading to enhanced mechanical properties in polymer composites. Varying the biochar filler loading can also affect the mechanical properties and stiffness of the polymer matrix, as observed through indentation testing. The high surface area of biochar can improve the reinforcement and mechanical interlocking of polymers, which can be predicted using the Halpin–Tsai–Nielsen and Verbeek models [43,150,151]. Higher biochar loads can also increase polymer matrix hardness, with biochar produced at higher operating temperatures generally resulting in higher surface area and improved mechanical strength and hardness of polymer composites [90].

### 6.7. Effects of Biomass Feedstock Properties on the Quality of Biochar

The choice of technology used to produce biochar has a significant impact on its effectiveness as a filler in polymer composites, as shown in experimental investigations. Factors such as the production operating temperature, the reactor design, and the feedstock used can affect the biochar’s porosity and mechanical properties [111]. Additionally, biochar derived from wood has been found to have higher mechanical and tensile strength when used as a reinforcement material. For instance, a composite made with Bael shell biochar at a 4 wt.% loading exhibited 180% higher tensile strength compared to virgin polymers [111]. Moreover, increasing the biochar filler percentage from 4% to 6% led to an increase in composite stiffness, hardness, and thermal resistance [46,47,152,153]. Biochar originating from pine wood, generated at a temperature of 500 °C and subsequently subjected to activation at 900 °C, has been employed in polypropylene composites to enhance their mechanical characteristics [9].

Residual biomass-derived biochar can enhance the thermal and acoustic properties of concrete, with thermal annealing of biochar up to 1500 °C further improving thermal properties [154]. When added to polymer composites, biochar can improve mechanical properties by facilitating stress release and transfer between filler and polymer composite [90]. Biochar’s stability, high electrical conductivity, low thermal conductivity, and high chemical stability and can reduce the flammability of polymer composites. Incorporating biochar in polymer composites decreases dependent thermal conductivity in composites, and the carbon content during carbonization affects thermal conductivity. For example, increasing the carbon content from 85 to 94 wt.% results in an increase in the order of magnitude of the bulk conductivity by 6 [18]. Compression of biochar increases electrical conductivity through the elastic behavior of electrically conductive biochar [18]. The porosity of biochar, which is affected by the pyrolysis operating temperature, heating rate, and biomass feedstock, also affects electrical conductivity. Higher operating temperatures result in biochar with higher electrical conductivity, with biochar produced from biomass pyrolysis at 700 °C exhibiting greater electrical conductivity due to higher surface area and lower porosity than that produced at 500–600 °C. Table 6 presents a summary of the factors which influence the properties of biochar.

### 6.8. Surface Interactions of Biochar Particles and Biochar Matrix in Polymer Composites

When biochar is added to the polymer matrix in the interfacial region, the surface morphology and viscoelastic properties of the polymer composite can be measured by Young’s modulus for the biochar–polymer composite. The addition of biochar decreases the polymer–polymer chain interactions and reduce the elastic properties of the polymer composite [30]. The selection of high pyrolysis temperatures produces more porous biochar depending on the feedstock type used; the characteristics of the biochar’s intra-pores change with different operating temperatures and residence times. Therefore, optimal charring temperatures and residence times should be specified to produce biochar with high intra-porosity [155]. The addition of biochar particles, assessed using nano-hardness values and Young’s modulus, have shown higher flexural strength up to 35% compared to conventional glass-fiber-reinforced polymers (GFRP); this also showed a lower Young’s modulus, which was reduced by 35% [133]. The surface morphology characteristics of the biochar were significantly impacted by both the source material and the temperature used during pyrolysis. The fibrous structures present in sawdust and furfural residue were preserved in their respective biochar samples, constituting the carbon framework within these biochar samples. Mesoporous biochar with a high surface area (1493 m^2^. g^−1^) is obtained at optimized conditions.

Functionalizing the surface of biochar can be achieved through the use of oxidizing agents like nitric acid, which increases the presence of oxygen surface-functional groups. While several studies have reported successful biochar surface functionalization with nitric acid, this process typically demands extended periods and the use of highly concentrated nitric acid. Moreover, this functionalization process tends to impact the morphology and pore structure of the modified carbon material, resulting in a reduction in the surface area. These reductions in surface area, coupled with the use of concentrated caustic chemicals and the time involved, present obstacles to implementing this functionalization process in industrial settings due to associated hazards and costs [156].

To address this issue, a functionalization process employing an autoclave is proposed, as these are readily available in palm oil mills for sterilization purposes. This method is suggested to expedite biochar functionalization, thereby enhancing biochar surface functionalities and, subsequently, its adsorption performance. The differentiating factor between the autoclave method and other reported modification techniques lies in the combination of elevated temperature and pressure during treatment. By offering multiple oxidation pathways, this approach is expected to significantly improve the efficiency of the process [157]. Addition of biochar improved the mechanical and thermal interactions of polymer composites in starch–biochar polymer matrixes. Hydrophobic biochar exhibits a positive water entry pressure, indicating that an external force is necessary to allow water to penetrate its intra-pores. In the absence of this external force, water cannot enter the intra-pores, leading to the prevention of intra-pore saturation and limiting the water-retention benefits of biochar [90]. Jeffery et al. noted that biochar derived from grass species did not enhance soil water retention, likely due to its high hydrophobicity (with an average contact angle of 118°). It is worth mentioning that grass biochar, while hydrophobic, still has lower hydrophobicity when compared to leaf or wood biochar. The degree of hydrophobicity in biochar varies depending on the production temperature and the feedstock used, but it is typically eliminated through brief exposure to the environment. Pre-treating biochar, either by initially wetting it or through composting, is likely to significantly mitigate issues associated with hydrophobicity [158].

### 6.9. Economic Feasibility of Biochar in Polymer Composites

The economic feasibility of using biochar in polymer composites depends on various factors, including the cost of biochar production, the performance improvement it offers to the composites, and the potential market demand for such eco-friendly materials [84]. These factors are described briefly in this section.

Biochar Production Costs: The cost of producing biochar can vary significantly depending on the feedstock used, the production method, and the scale of production. Commonly recommended feedstocks for biochar production include agricultural residues, wood waste, and organic waste materials. Generally, biochar may cost between USD 222 and USD 584 per ton to produce, deliver, and spread on fields [159]. However, the cost associated with transportation of biochar, the related equipment purchase, thermochemical conversion process, and high dosage use makes agricultural and environmental uses of biocarbon infeasible [160]. Therefore, it is crucial to assess the local availability of feedstock and the efficiency of the production process to determine the cost of biochar.

Performance Enhancement: Biochar can be added to polymer composites to enhance their mechanical, thermal, and environmental properties. This enhancement may reduce the amount of expensive polymer material required to achieve the same performance, potentially offsetting the cost of biochar. However, the extent of performance improvement and the resulting reduction in polymer usage should be evaluated in terms of their economic impacts.

Market Demand: The global market demand for biochar-based polymer composites is a critical factor. If there is a growing trend toward sustainable and eco-friendly materials in various industries (e.g., construction, automotive, and packaging industries), then it might create a favorable market for biochar composites. The willingness of consumers and businesses to be adaptable and to potentially pay a premium for eco-friendly products can also affect the economic feasibility.

Regulatory and Certification Costs: Depending on the region and industry, there may be regulatory requirements or certifications associated with the use of biochar in polymer composites. Compliance with these standards may add to the overall cost of production and should be considered in economic feasibility assessments.

Competitive Pricing: To determine economic feasibility, it is essential to compare the cost of biochar-based polymer composites to existing alternatives. If the price of traditional polymer composites is significantly lower, it may be challenging to gain market traction unless there are other compelling benefits. However, several countries have started to offer carbon tax benefits to industries producing and utilizing biocarbon products as an incentive for reducing their carbon footprints; accordingly, the reduction in costs pertaining to biochar-based polymer composites and the enhancement of their properties should be considered.

Long-term Benefits: The long-term benefits of using biochar, such as potential cost savings from reduced polymer usage, extended product lifespan, and lower environmental impact should be considered. These factors can positively influence economic feasibility over time, which is a factor which could be used to encourage consumer adaptability.

Research and Development Costs: There may be research and development costs to consider when assessing the costs related to the development of novel biochar-based polymer composites. These costs can impact initial economic feasibility assessments, but may lead to competitive advantages in the future.

Considering aforementioned factors, the economic feasibility of using biochar in polymer composites is a complex issue that depends on multiple factors; it is essential to determine whether the use of biochar in polymer composites makes economic sense in a specific application or industry. Additionally, staying informed about evolving market trends and consumer preferences for sustainable materials is crucial for making informed decisions [158,159,160].

## 7. Research Gap and Future Prospective

Biochar is a sustainable and organic filler that can enhance the properties of polymer matrices. However, there is a research gap in several aspects of biochar usage as a re-enforcement material. To develop biochar with optimal mechanical properties and electrical conductivity, high thermal control is necessary for pyrolizers. Further studies are required to analyze the improvement of mechanical, thermal, and acoustic properties at different biochar loads and investigate the impact of biochar particle size and morphology on the mechanical properties of polymer composites. The optimal biochar loadings that increase the glass transition temperature and the thermal stability of composites need to be investigated. Moreover, the morphology of biochar requires investigation to deter-mine its effects on stiffness and fire-resistant properties. The optimal biochar loads that achieve high storage modulus and better dispersion in a polymer matrix need to be determined. The effects of biochar loading that improve flame retardancy properties are also yet to be investigated. Future research topics include the development of pyrolizers that can achieve high thermal control and control over biochar particle size and morphology. Furthermore, understanding how filler morphology affects biochar properties and its impact on the electrical conductivity of polymer composites is crucial. Ultimately, further studies are necessary to optimize the use of biochar as a sustainable and effective filler in polymer composites.

## 8. Conclusions

Biochar has emerged as a promising additive for polymer composites, offering significant improvements for their thermal, mechanical, and electrical properties. Compared to other fillers, biochar demonstrates superior thermal stability. It is lightweight, easy to fabricate, and cost-effective, making it an attractive option for weight reduction in automobiles. The addition of ions to biochar further enhances the electrical conductivity of polymer composites. To achieve the optimal improvement in mechanical properties, a 10 wt.% biochar addition is recommended; as biochar content increases beyond this threshold, the mechanical properties of polymer composites tend to deteriorate. For biochar to be effective, it should possess high surface area and porosity, high cation exchange capacity (CEC), neutral–high PH, and a high carbon mass ratio. Optimal biochar properties and surface area are achieved at higher operating temperatures during pyrolysis and carbonization. Biochar produced at temperatures above 1000 °C demonstrates higher electrical conductivity. Biochar compression also increases electrical conductivity in polymer composites. Studies are necessary to evaluate the toxicity and the thermal and mechanical stability of biochar in various polymer composites, and to assess its suitability for diverse industrial applications. Overall, biochar shows promise as a sustainable organic filler with exceptional physical properties.

## Figures and Tables

**Table 1:** Typical mechanical properties of different types of carbon fibers.

Carbon Fiber	Fiber Mechanical Strength	Elasticity Modulus (GPa)	Tensile Strength (MPa)	Ultimate Strain (%)	References
Pan-based fiber	High modulus	300	5000	1.73	[67,72]
Hysol Grafil Apollo	Ultra-high modulus material	450	3500	0.77	
Pitch-based fiber	High-quality fiber	227	2758	1.76	
Hysol Union Carbide	High modulus	240	3440	1.75	

**Table 2:** Comparison of composites reinforced with biochar, CNT, and carbon filaments.

Filler Material	Biochar	CNT	Carbon Filaments	References
Raw material and its production method	Synthesized by pyrolysis above 500 °C.	Produced by either catalytic chemical disposition, laser ablation, or arc discharge.	Carbon fibers are converted to long strands and heated to very high temperatures without contact with oxygen.	[11,43,67,68,73]
Advantage of filler material	Improves thermal stability. Addition of biochar between 20 wt.% and 30 wt.% increases the glass transition temperature of the polymer composite by 7–20 wt.%.	CNT possess excellent adsorption ability due to strong interaction with heavy metal ions.	Film and rigid mechanical structure; high tensile strength and stiffness; high chemical and heat resistance.	
Disadvantage of filler material	The properties of biochar depend upon properties of biomass and thermal conditions during pyrolysis. Different biochar yields different mechanical properties.	Homogenous dispersion of CNTs is difficult especially at high loadings. Molecular modification is needed to enhance dispersion and reduce aggregation. Also, they have a very high cost compared to alternative fillers.	High production cost; negative temperature coefficient of resistance.	

**Table 3:** Expected biochar and hydrocarbon product yields from biomass pyrolysis at different heating rates.

Pyrolysis	Vapor Residence Time (s)	Heating Rate (°C)	Process Temperature (°C)	Solid Yield (wt.%)	Liquid Yield (wt.%)	Gaseous Yield (wt.%)	References
Slow	450–500	0.1–1	300–700	~35	~30	~35	[21,89,108]
Fast	1–10	10–200	400–800	~20	~50	~30	[45,46,108]
Flash	<1	~1000	800–1000	~12	~12	~75	[20,21,108]

**Table 4:** Polymer composites prepared with different biochar sources, loads, and fabrication methods.

Polymer	Biochar Source	Filler Loading Rate	Carbonization Temperature (°C)	Fabrication Method	References
High molecular polyethylene	Bamboo tree	5 to 9 wt.%	1000	Compression molding	[12,13]
Polyethylene	Biochar synthesized from wood (50 to 100 µm) (MAPP) Biochar (1000 µm and 50 µm)wood	24 to 30 wt.%	900	Extrusion and injection molding	
Polypropylene	Miscanthus grass (106–125 and <20 µm)	NR	630	Melt processing	[24,25,125]
Nylon 6	Miscanthus fiber	20 wt.%	500 and 900	Melt compounding and injection molding	
Polypropylene	Landfill pine wood	24 wt.%	900	Extrusion and injection molding	[40,41,42,43]
Polylactic acid	Mixed hardwood (<250 μm)	2, 6 and 10 wt.%	800	Solvent casting	
HDPE	Rice husk	30–70 wt.%	500	Twin-screw extrusion	[90]
PLA	Waste brewed coffee powder	1, 2.5, 5, and 7.5 wt.%	700	Solvent casting	[123]
Polypropylene	Date palm waste	0–15 wt.%	700 and 900	Micro injection molding	[124]
Polypropylene	Pine wood	6, 12, 18, 24, and 30 wt.%	450	Twin-screw extrusion and melt blending	[126]
Polylactic acid and BIOPLAST GS2189	Wood residue and sewage sludge	0, 10, 20 wt.%	550	Injection molding	[127]
Polyester	Cashew nutshell waste	5, 10, 15 wt.%	500	Resin casting	[128]
Polypropylene	Landfill pinewood waste	0, 15, 25, 30, 35 wt.%	Pyrolyzed at 500 followed by activation at 900	Extrusion and injection molding	[129]
HDPE	Rice husk	10–70 wt.%	600	Micro twin-screw extruder	[130]
Polylactic acid	Grapevine (120 mesh)	1–10 wt.%	500	Compression molding	[131]
Polylactic acid	Bamboo waste biochar, aramid fiber, and silica	0–40, 0–20, and 0–20 wt.%, respectively	500	Micro-injection molding	[132]
Fiber glass reinforced epoxy resin	Spruce wood pellets	0, 5, and 10 wt.%	150 °C for 30 min followed by 450 °C for 30 min	Resin casting	[133]
Epoxy	Arhar stalks and Bael shell	2, 4, and 6 wt.%	800	Hand lay-up technique	[111]
Polypropylene	Landfill pine sawdust biochar and wool	0–25, and 0–10 wt.%, respectively	500 followed by activation at 900	Twin-screw extrusion	[134]
Low viscosity epoxy resin	Maple tree (10 μm)	0, 1, 2, 4, and 20 wt.%	600 and 1000	Resin molding	[135]
Diglycidyl ether of bisphenol A based epoxy resin	Coconut shell biochar and *C. urens* fibers	1–7, and 30 vol.%, respectively	RT-200 followed by 200–500 followed by 500–800	Hand lay-up technique	[136]

**Table 5:** Different polymer filler materials and changes in properties as well as a preparation method.

Polymer Type	Polymer Blend	Plasticizer/Hardener/ Stabilizer/Compatibilizer	Preparation Method	Change in Properties	References
Polystyrene	Single-wall carbon nanotubes	Dimethyl; dibutyl	Catalytic chemical vapor deposition for CNTs; copolymerization for composite	Glass temperature (Tg) increase by 3 °C	[139]
Poly(propylene carbonate)	Nanoclay at 4 wt.%	Glass fiber; talc or calcium carbonate	Co-polymerization	Glass temperature (Tg) increase by 13 °C	[25,26,139]
Polylactide	Naoclay at 3 wt.%		Co-polymerization of CO_2_ and chemicals called epoxides	Glass temperature decreases by 4 °C	[55,67,68,69,70,71,73,74,75,76,77,78,79,80,81,82,83,84,85,86,87,88,89,90,91,92,93,94,95,140,141]
Rubber	Nanoclay at 5 wt.%	Adipates and Sebacates	Triethyl citrate or tributyl citrate	Glass temperature increases by 3 °C	[94,95,96,97,98,99,100,101,102,103,104]
Glass-fiber-reinforced composites	Biochar at 5 wt.% to 10 wt.%	Organic esters	Pyrolysis and carbonization for biochar; extrusion for polymer composite	Higher glass temperature; higher stiffness and fire retardancy	[12,13,14,84,142,143]
Poly(dimethyl siloxane)	Silica pore size between 2 nm and 3 nm	Trimethylsiloxy-terminated	Replica molding or casting	Higher glass temperature at 10 °C	[12,13,14,84,142,143]
Unsaturated polyester resin	High-purity silica at 30% (average particle size = 3, 6, 16, and 22 µm), 10% (average particle size = 0.1, 1, 2, 3, 6, 16, and 22 µm), 12%, 16%, 24%, 37%, and 46% filler volume	methyl-ethyl-ketone-peroxide (MEKPO)	Molding or casting	Present good homogeneous and effective bond between matrix and particles; hardness and elastic modulus increases linearly with filler content	[144]
Unsaturated polyester	Rice husks at 10, 15, 20, and 25 wt.%	methyl-ethyl-ketone-peroxide (MEKPO)	Room-temperature molding	Tensile strength of the composite decreases with increased filler loading; Young’s modulus increased remarkably for 15 wt.%; the composite showed higher water absorption	[145]
Semi-crystalline LDPE, and two amorphous (polystyrene and polymethyl methacrylate)	Graphene-based flakes (average flake size = 1.45 μm); fumed silica (average particle size = 0.2–0.3 μm)		Graphene-based flakes are prepared using micro-fluidization; polymer composite using extrusion melt-mixing followed by injection molding	Lower filler content increases the viscosity; glass transition temperature showed mixed behavior, e.g., increasing/decreasing nature	[91]
Epoxy belong to diglycidyl ether of bisphenol A (DGEBA) family	Potassium titanate whisker (PTW) with aspect ratio 20–40	Amino based	Mechanical stirring and vacuum-assisted casting method	15 wt.% composite showed improved wear resistance and highest friction coefficient; positive effect on hardness, density, and stiffness. But the strength and ductility decreases	[146]
Polyvinyl chloride (PVC) of density 1.41 g/cm^3^	Calcium Carbonate (CaCO_3_) in 1–2 μm particle size	Arkopan	Melt-blended in twin-screw extruder	Increases glass transition temperature, storage, and elastic modulus; however, reduces tensile strength and elongation at break and no significant effect on water absorption	[147]
High-density polyethylene (HDPE) of different grades	Poplar wood flour sieved through 60 mesh	Maleic anhydride-modified polyethylene with melt index 6 g/10 min, and a graft ratio of 1.1%	Twin-screw extrusion	Increased filler content improves filler–filler interaction; agglomeration of wood particles which increases storage modulus and viscosity of the composite with decreased tensile strength; elongation at break; notched impact strength due to stress concentration	[148]
Glass fibers reinforced low-temperature curing epoxy	Flyash (Class-F) with density 2 g/cc and total silica and alumina content > 70%	Hardener HY951	Resin molding	Steep increase in impact strength is noted for small filler content; compressive strength found to be decreasing	[149]
Visible-light-activated polyphenylene polymethacrylate resin	Silanated barium borosilicate with particle size 2 µm (at 20, 40, 45, 50, and 53 vol% loading) and 15 µm (at 20, 40, 50, 60, and 65 vol% loading)			Increased filler level enhances compressive strength, hardness, stiffness with reduced water absorption; all the resins showed significant improvement in resistance to wear by hydroxyapatite than unfilled resins; however, particle sizes of filler have moderate impact on the resin properties	[43]

**Table 6:** Summary of influential factors and their impact on biochar product yield.

Influential Factor	Description	Biochar Product Yield	References
Lignin weight content of biomass feedstock	Lignin content has an impact on biochar yield and fixed carbon content	High biochar yield based on lignin content with low surface area; high surface PH and functional groups; high ion exchange capacity.	[2,22,71,76,78,86]
Influence of inorganic content	Inorganic content weight percentage has an impact on biochar properties	The inorganic content increases, biochar yield through bond dissociation energy between organic and inorganic carbon increases; higher electrical conductivity and loss of volatile matter.	
Higher operating temperature in thermochemical reactions	Low product yield of biochar; high fixed carbon and lower volatile matter	Higher surface area of biochar and higher pore volume with stronger mechanical properties; higher PH and electrical conductivity; high crushing and impact strengths of biochar.	

## Data Availability

Not applicable.

## Glossary

Symbols	
$H_{m}$	Sample melting enthalpy
Pa-s	Pascal second (Pa-s)
S/cm	Siemens per centimeter
TPA	Tera pascal (elastic modulus)
Abbreviations	
CFRP	Carbon-fiber-reinforced polymer
MWCNTs	Multi-walled carbon nanotubes
